# Carcinoma of the uncinate process of the pancreas presenting with deep vein thrombosis: a case report

**DOI:** 10.4076/1757-1626-2-8780

**Published:** 2009-09-16

**Authors:** Kumaran Shanmugarajah, Elaine Hui, Nikhil Vergis, Chris Schelvan, Stephen Robinson

**Affiliations:** 1 Department of Endocrinology and Metabolic Medicine, The Mint Wing, Imperial College Healthcare NHS Trust, St Mary's Hospital, London, W2 1NY, UK

## Abstract

The uncinate process is a hook-like projection of the inferior aspect of the head of the pancreas. Carcinoma of the uncinate process of the pancreas is considered to be rare, difficult to diagnose and particularly devastating. The current method of detection is computed tomography. We report a case of carcinoma of the uncinate process of the pancreas in a patient who initially presented with deep vein thrombosis. The diagnosis of carcinoma of the uncinate process of the pancreas should be considered in patients who present with primary thromboembolic disease and other nonspecific signs.

## Introduction

The head of the pancreas sits within the C-shaped curve of the duodenum. The uncinate process is an extension of the inferior part of the head of the pancreas that projects medially and wraps around the superior mesenteric vessels. Pancreatic carcinoma within the uncinate process appears to be rare, with previous reports describing an incidence of 2.5 percent (3 of 119 patients) [1], 8 percent (39 of 506 patients) [2], and 10.7 percent (6 of 56 patients) [3], of pancreatic malignancies. We report a patient with carcinoma of the uncinate process of the pancreas presenting with venous thrombosis, a rare case with an important presentation.

## Case presentation

A 59-year-old British, Caucasian male presented with a one week history of left lower leg pain. The pain was associated with warmth and mild swelling. There was no history of trauma or prolonged immobility, and there was no associated chest pain. The patient reported a weight loss of 10 lbs over the previous 2 months, with anorexia over the previous month. He had been drinking 60 units of alcohol weekly for many years and was a life-long non-smoker. On physical examination, the patient was well hydrated and apyrexial. Cardiovascular examination revealed a systolic murmur. His abdomen was soft, non-tender with the splenic tip palpable in the left upper quadrant.

Laboratory investigations revealed a raised D-dimer. In addition, there was an elevated C-reactive protein, erythrocyte sedimentation rate and white cell count. Vascular studies of the left leg demonstrated thrombus in the posterior tibial and peroneal veins below the knee and superficial thrombophlebitis in the long saphenous vein.

**Figure 1:**
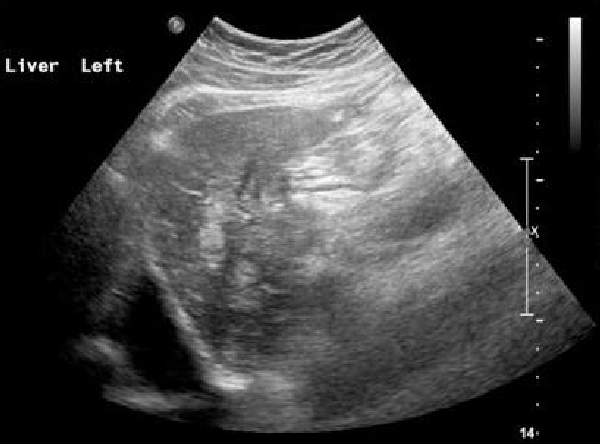
**Ultrasound scan of the abdomen, demonstrating multiple echogenic foci within the liver**.

**Figure 2:**
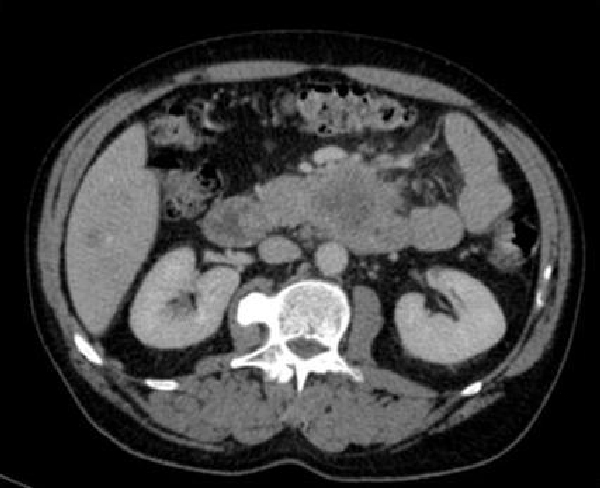
**CT scan demonstrated a mass, arising from the uncinate process of the pancreas**.

The patient was admitted and treatment was commenced for his deep vein thrombosis. Echocardiography demonstrated calcific aortic stenosis and no valvular vegetations. During his admission, the patient developed a thrombus in a right brachial vein and his liver function tests became elevated. A subsequent ultrasound scan of the abdomen revealed multiple echogenic foci within the liver (Figure 1). CT scan of the abdomen demonstrated a large mass arising from the uncinate process of the pancreas measuring 56 × 50 mm, with associated invasion into the superior mesenteric vein (Figure 2). Liver histology confirmed poorly differentiated adenocarcinoma with morphology consistent with a pancreatic primary. Although chemotherapy was considered at the hepatobiliary multidisciplinary meeting, his condition deteriorated rapidly and he subsequently passed away a few weeks after discharge.

## Discussion

Patients with carcinoma of the uncinate process of the pancreas exhibit nonspecific symptoms with weight loss and upper abdominal pain as the most significant ones [2,4]. Importantly, despite the uncinate process being part of the head of the pancreas, jaundice does not appear to be an early symptom [1,2,4]. Furthermore, the close anatomical relationship between the uncinate process and the superior mesenteric vessels results in early vascular involvement in these cancers.

In carcinoma of the uncinate process of the pancreas, CT scan is believed to be the best diagnostic tool [2,4]. Due to the increased distance of the common bile duct and pancreatic duct from the uncinate process, Endoscopic Retrograde Cholangio-Pancreatography is of little use [2,4]. Transcutaneous ultrasound has an overall sensitivity of 70-80 percent in the diagnosis of pancreatic carcinoma [5]. However, due to the anatomical position of the uncinate process, ultrasound imaging of this area is more likely to be obscured by overlying bowel [2]. Indeed, in our patient the pancreas was obscured by bowel gas on ultrasound imaging of the abdomen. CA19-9 is a valuable tool and its sensitivity and specificity has been demonstrated at over 80% [6]. In patients with carcinoma of the uncinate process, its sensitivity has been documented at 90.4 percent^4^ and its specificity at 96 percent [2].

Pancreatic carcinoma is regarded as a devastating disease. Due to the aforementioned nonspecific signs and early vascular involvement, malignancy of the uncinate process is particularly lethal. Birk et al were able to demonstrate that the median survival of carcinoma of the uncinate process was significantly lower than carcinomas affecting other areas in the head of the pancreas (5 months versus 11 months) [2].

In our patient, venous thrombosis was the first manifestation of pancreatic carcinoma. The relationship between thromboembolic disease and pancreatic carcinoma was first described by Sproul in 1938 [7], and is now well documented. Indeed, the incidence of thromboembolic disease in patients with pancreatic carcinoma has been estimated as being as high as 57% [8]. In an analysis of 66,000 patients with cancer and neutropenia, it was discovered that those patients with pancreatic carcinoma had the highest risk of venous thromboembolic disease [9]. This relationship can be explained by the generation of an intrinsic hypercoagulable state in pancreatic carcinoma, which seems to be related to enhanced tumour growth and angiogenesis [10]. While thromboembolic disease is often the complication of an underlying malignancy, this case highlights that it may be the presenting feature in carcinoma of the uncinate process of the pancreas.

## Conclusion

Carcinoma of the uncinate process of the pancreas is a disease with a horrific prognosis. Furthermore, its nonspecific nature means that the diagnosis is often missed. Therefore, in patients presenting with primary thromboembolism and other nonspecific symptoms, carcinoma of the uncinate process of the pancreas must be considered. The CA19-9 level should be measured and if raised, a prompt CT scan should be performed.

## Consent

Written informed consent was obtained from the patient's next of kin for publication of this case report and accompanying images. A copy of the written consent is available for review by the Editor-in-Chief of this journal.

## Competing interests

The authors declare that they have no competing interests.

## Authors' contributions

CS analysed and interpreted the radiological data regarding the diagnosis of carcinoma of the uncinate process of the pancreas. KS, EH, NV and SR were the major contributors in writing the manuscript. All authors read and approved the final manuscript.
